# Current and Emerging Assays for Measuring Human T-Cell Responses Against Beta-Cell Antigens in Type 1 Diabetes

**DOI:** 10.3390/biom15030384

**Published:** 2025-03-06

**Authors:** Ting-Chen Lin, Matthew Lacorcia, Stuart I. Mannering

**Affiliations:** Immunology and Diabetes Unit, St. Vincent’s Institute of Medical Research, Fitzroy, VIC 3065, Australia; ting-chen.lin@svi.edu.au (T.-C.L.); mlacorcia@svi.edu.au (M.L.)

**Keywords:** type 1 diabetes, CD4^+^, CD8^+^, antigen, proinsulin, C-peptide, T-cell assay

## Abstract

Type 1 diabetes (T1D) is an autoimmune disease caused by T-cell mediated destruction of the pancreatic insulin-producing beta cells. Currently, the development of autoantibodies is the only measure of beta-cell autoimmunity used in the clinic. Despite T-cells’ well-accepted role in the autoimmune pathogenesis of human T1D, autoimmune T-cell responses against beta cells remain very difficult to measure. An assay capable of measuring beta-cell antigen-specific T-cell responses has been a long-sought goal. Such an assay would facilitate the direct monitoring of T1D-associated T-cell responses facilitating, earlier diagnosis and rapid evaluation of candidate immune therapies in clinical trials. In addition, a simple and robust assay for beta-cell antigen-specific T-cell responses would be a powerful tool for dissecting the autoimmune pathogenesis of human T1D. Here, we review the challenges associated with measuring beta-cell antigen-specific T-cell responses, the current assays which are used to achieve this and, finally, we discuss BASTA, a promising emerging assay for measuring human beta-cell antigen-specific CD4^+^ T-cell responses.

## 1. Introduction

Type 1 diabetes (T1D) is a tissue-specific autoimmune disease [1]. It is caused by the T-cell-mediated destruction of the pancreatic insulin-producing beta cells. T1D typically develops within the first decades of life, although it can also be diagnosed well into adulthood [2]. An ongoing, albeit asymptomatic, autoimmune beta-cell specific T-cell response persists until an individual develops clinical T1D [3]. Currently, T1D is incurable, and people with T1D must receive regular exogenous insulin injections to maintain their glucose homeostasis [4].

Autoantibodies specific for beta-cell proteins arise prior to the onset of clinical T1D [3]. While antibodies are not believed to contribute directly to the autoimmune pathogenesis of T1D [5,6,7], they are a useful marker of ongoing, otherwise silent, beta-cell-specific autoimmune T-cell responses. Currently, autoantibodies that bind to the beta-cell proteins: insulin, glutamic acid decarboxylase 65 kDa (GAD65), insulinoma antigen 2 (IA-2) and Zinc Transporter 8 [8,9] are most frequently used clinically.

The progression of T1D has been classified into three stages [3]. Stage 1 is characterized by the presence of two or more islet autoantibodies but with no evidence of dysregulated glucose homeostasis [3,10]. In stage 2, autoantibodies persist, but dysglycemia can be detected with an oral glucose tolerance test (OGTT). Despite this, individuals remain asymptomatic [3,10]. Stages 1 and 2 of T1D can last for months to decades [3]. Stage 3 is marked by the onset of clinical T1D [10]. Individuals have symptomatic hyperglycemia due to significant beta-cell loss, rendering the individual unable to maintain glucose homeostasis [3,10]. Symptoms of T1D include frequent urination, weight loss, blurred vision and excessive thirst and hunger [11]. Complications such as diabetic ketoacidosis may also occur when the high glucose level and low insulin level lead to the breakdown of fats, increasing the body’s ketone concentration [12]. T1D is most frequently diagnosed at stage 3 T1D [11,13]. Individuals with long-standing clinical T1D are sometimes referred to as being in stage 4 T1D.

An individual’s risk of developing T1D is determined by both their genetics and environment [14]. The genetic loci which modify an individual’s risk of developing T1D have been well characterized [15]. Notably, of the genetic loci, the HLA region, particularly class II HLA, has the greatest impact on an individual’s risk of developing T1D [16]. This genetic association provides further evidence for the role of CD4^+^ T cells in the autoimmune pathogenesis of T1D.

## 2. Measuring Antigen-Specific Autoimmune Responses in T1D

### 2.1. Why Measure Beta-Cell Antigen-Specific T-Cell Responses in T1D?

Autoantibodies against beta-cell proteins are the only measure of autoimmunity currently available to clinicians. However, it is now clear that T cells, not antibodies, mediate the destruction of the beta cells in T1D [7,17]. While several assays have been developed that can detect beta-cell antigen-specific T-cell responses in human blood samples (see below), none are sufficiently simple and robust to be suitable for routine clinical analysis of T-cell responses in a similar way to autoantibodies. A simple and robust assay capable of measuring the beta-cell antigen-specific T-cell response, referred to as ‘a T-cell assay’, is required for three broad applications [18]. These are each discussed below.

First, a T-cell assay is required to improve the diagnosis of T1D. Currently, monitoring of beta-cell autoimmunity relies solely on autoantibodies [19,20]. While there is now considerable clinical and laboratory expertise in measuring and interpreting beta-cell autoantibody results [21,22], they still give little insight into when an individual will progress to stage 3 T1D. The number of specificities an individual has autoantibodies against is directly proportional to their risk of developing T1D, but the time period is long [23]. This gives little useful guidance to clinicians and, in the absence of an approved preventative therapy, gives parents further opportunities for anxiety.

Second, a T-cell assay is required to support clinical trials of experimental immune modulatory therapies to prevent or reverse T1D. Currently, clinical trialists must rely on metabolic end-points to determine if the therapeutic agent they are testing is beneficial [24]. Consequently, the trials must run for many years to allow a sufficient number of participants in the control group to progress far enough for a conclusion to be drawn on the agent’s efficacy, or lack thereof. A T-cell assay capable of directly measuring T-cell responses against beta-cell antigens could serve as a surrogate marker for the agent’s efficacy. Clear evidence that the putative pathogenic T-cell response had been modified in some way would be strong evidence that the agent was likely to be beneficial in preventing or reversing T1D. Conversely, clear evidence that an agent was not having an impact on beta-cell antigen-specific T-cell responses would focus attention on other, more promising agents. If an appropriate T-cell assay was available, the duration of clinical trials could be dramatically reduced. This would reduce both the cost and the burden on participants of a trial.

The third broad area of application for a T-cell assay is in research. Despite decades of intense research, the autoimmune basis of human T1D remains poorly understood. The difficulty in analyzing beta-cell antigen-specific T-cell responses has made dissecting autoimmune responses in T1D particularly difficult [25]. As we discuss below, several assays have been developed that can detect beta-cell antigen-specific T-cell responses. However, each assay has its limitations.

### 2.2. What Are the Challenges in Measuring Beta-Cell Antigen-Specific T-Cell Responses?

Why is it so difficult to measure beta-cell antigen-specific T-cell responses in T1D? Broadly speaking, there are three major challenges. First, autoantigen-specific T cells are present at very low frequencies in peripheral blood, which makes them very difficult to detect [18,25]. This means that relatively large volumes of blood must be collected and sophisticated approaches must be used to distinguish the very few beta-cell antigen-specific T cells from the very large number of irrelevant bystanders. Second, despite considerable progress, it is still not clear which antigens [26,27,28], and epitopes derived from them, are ‘seen’ by disease-associated autoreactive T cells. Our knowledge of clinically relevant antigens and epitopes has grown rapidly over the past ten years [26,29]. We now have a bewildering array of potential epitopes, creating a problem of distinguishing the useful and relevant from the merely interesting. Finally, in marked contrast to responses to infectious agents, T-cell responses to autoantigens are weak. This adds to the challenge of identifying these responses [30]. This complexity is further compounded by the fact that the presence of autoreactive T cells in the blood can be non-disease-specific [31,32]. These factors collectively hinder the accurate measurement of antigen-specific T-cell responses, making it hard to understand the autoimmune mechanisms underlying T1D and develop effective immune monitoring assays and immunotherapies.

### 2.3. What Are the Features of the Ideal T-Cell Assay for Measuring Beta-Cell Antigen-Specific T-Cell Responses?

The features of an ideal T-cell assay clearly depend upon the application and the reason for performing the assay. Having said that, there are some common features that are desirable independently of the reason for performing the assay. The ability to collect a sample in a relatively non-invasive manner is essential. One could argue that sampling T cells that infiltrate the islets [33,34] would give direct insights into the T-cell responses occurring at the site of autoimmunity, but this is not possible because of the risk to the individual. In practice, peripheral blood is the only tissue available for routine sampling. The volume of blood required is a practical problem for many T-cell assays. The ideal assay would require a very small volume of blood. However, given the low frequency of responding T cells [35], T-cell assays will always require more blood than antibody tests. The ideal T-cell assay would be very sensitive, with the capacity to detect weakly responding rare T cells. Our growing knowledge of the antigens and epitopes seen by T1D-associated T cells has provided us with a growing list of candidate antigens [26,36]. Hence, a T-cell assay needs to be able to accommodate multiple antigens, ideally as a pool or individual peptides or proteins. An ideal T-cell assay would be very consistent, with a low intra- and inter-assay variability. Again, the low frequency of responding cells and the need to keep blood volumes low make this a particular challenge for T-cell assays. An ideal assay should be very simple to perform, and for many applications, an assay that can be carried out close to the point of care would be advantageous. Finally, an ideal assay would be logistically simple. That is, samples would be easily collected, stored, shipped and analyzed. With these ideals in mind, we discuss the strengths and weaknesses of the assays currently in use.

## 3. Current Assays for Measuring Human Beta-Cell Antigen-Specific T-Cell Responses in T1D

Several assays capable of measuring human T-cell responses against beta-cell antigens have been developed [18]. These assays, and a synopsis of their strengths and weaknesses, are summarized in Table 1. The major classes of T-cell assays currently in use include (i) the CFSE-based (5,6-carboxyfluorescein diacetate succinimidyl ester) proliferation assay [37,38,39], (ii) IFNγ enzyme-linked immunosorbent spot (ELISpot) assay [40], (iii) peptide/HLA-tetramer-based assay [41] and (iv) activation-induced marker (AIM) assays [42].

### 3.1. The CFSE-Based Proliferation Assay

Assay: The CFSE-based proliferation assay is used to detect rare antigen-specific T cells by their proliferation in response to an antigen [37]. By labelling all cells with the fluorescent dye CFSE (or similar dyes), cells that proliferate can be identified by their dilution of the dye with each cell division cycle. This allows the few cells that proliferate to be distinguished from the many bystanders that do not proliferate. Over the last twenty years, dye-dilution proliferation assays have largely overtaken measuring proliferation via uptake of ^3^H-thymidine because they are non-radioactive, more sensitive and allow for the lineage (CD4^+^, CD8^+^, etc.) of the responding cells to be defined directly [43].

Advantages: The CFSE-based proliferation assay is very sensitive. It is relatively easy to perform and has a low cost [44]. Additionally, the assay counts the number of cells that have proliferated in response to an antigen, which allows for the proliferation to be quantified. The proliferation of any cell lineage can be determined by changing the monoclonal antibodies used to stain the cells at the conclusion of the experiment. The antigen-responsive cells can be isolated for further analysis or cloning [45]. Labelling cells with stable fluorescent dyes, such as CFSE, is a versatile platform for identifying antigen-responsive cells. This can be used to isolate antigen-responsive cells for downstream applications, such as cloning, cell surface marker expression, cytokine production or transcript profiling [45].

Disadvantages: Each sample must be processed and analyzed by flow cytometry individually, making it unsuitable for high-throughput clinical settings. Because the cells need time to proliferate, the assay takes a week to perform. Moreover, we have recently shown that many of the cells that proliferate are not specific for the antigen of interest [39]. This is particularly true for antigens, such as autoantigens, which stimulate weak CD4^+^ T-cell responses. This observation complicates the analysis of antigen-responsive cells in the CFSE assay, as it is unknown, without using other approaches, which cells are antigen-specific.

Applications: The CFSE assay is ideal for detecting the proliferation of rare antigen-specific T cells [18]. It is also ideal for enriching viable proliferating antigen-specific T cells [45].

### 3.2. ELISpot Assay

Assay: The ELISpot assay works by capturing a cytokine, usually interferon-gamma (IFN-γ) produced by T cells upon antigen-driven activation [40]. The cytokine detection is similar to an ELISA, but it uses a substrate that produces an insoluble product, which, in turn, creates a visible halo around each cytokine-producing cell. These spots can be counted to quantify the response to the antigen [46].

Advantages: The ELISpot assay is very sensitive; it is able to detect antigen-specific T cells at low frequencies, as low as 0.8–80 within 10,000 T-cells [25]. The ELISpot assay provides both quantitative and functional data in the form of spot counts of a particular cytokine, which correlate directly with the number of cytokine-producing cells [47]. Advances in ELISpot, such as fluorescent detection, (i.e., Fluorospot assays), allow for the simultaneous measurement of multiple cytokines [48,49].

Disadvantages: Although it is excellent for measuring the production of a single cytokine, the ELISpot assay does not provide detailed functional information on other aspects of T-cell activity, such as cytotoxicity or proliferation. Furthermore, it is difficult to phenotype the cytokine-secreting cells while measuring cytokine production [50]. Usually, it only measures a single cytokine, so cells that do not make that cytokine are not detected.

Applications: The ELISpot assay is ideal for detecting cytokine-producing cells present at low frequencies in peripheral blood.

### 3.3. pHLA-Tetramer-Based Assays

Assay: The tetramer-based assay is used to detect antigen-specific T cells. This is achieved by staining antigen-specific T-cell receptors (TCRs) with fluorescently labelled pMHC/HLA complexes [41]. HLA–class I/peptide complexes are most frequently used to detect antigen-specific CD8^+^ T cells, but CD4^+^ T-cell responses can be detected using HLA–class II/peptide complexes.

Advantages: The tetramer-based assay is highly antigen-specific, enabling validation of selected epitopes [25]. It is also quantitative [41,51]. Furthermore, it allows for the surface markers expressed by antigen-specific T cells to be determined by FACS. Lastly, as long as the cells are kept alive, they can be recovered for further culture or analysis.

Disadvantages: The major disadvantage of tetramer-based approaches is that they require detailed knowledge of both the epitope and its HLA restriction. A custom-made peptide/MHC complex is required for each epitope/HLA combination to be studied. Because the tetramer binding relies on cognate TCR-pHLA interactions, the HLA genotype of the individual who gives the sample needs to be known. The assay also does not work well if the peptide binds weakly to the HLA/MHC. Some protocols covalently link the peptide to the HLA [52]. Moreover, it is also labor-intensive and technically demanding, which can be unsuitable for routinely monitoring T-cell response in a clinical setting.

Applications: The assay is ideal for detecting and characterizing antigen-specific T cells with high specificity. It can also sort antigen-specific T cells for TCR sequencing, which is useful in validating specific epitopes.

### 3.4. The AIM Assay

Assay: The activation-induced marker, or AIM, assay identifies antigen-specific T cells based on the upregulation of cell surface markers following T-cell receptor stimulation by antigens detected by flow cytometry [53]. A larger panel of antibodies can be used to gain more detailed insight into the phenotype of the responding cells [44].

Advantages: The AIM assay detects rare, antigen-specific T cells directly following a brief in vitro activation. AIM assays can detect responses to a wide array of antigenic targets [54], including whole antigens and synthetic peptides in single or pooled formats [44]. The responding cells can be purified by sorting and subjected to further investigation.

Disadvantages: It can exhibit a lack of sensitivity due to variable background responses, which can obscure true antigen-specific signals [44]. The assay can require complex and sometimes subjective data interpretation, particularly when the markers show a modest shift in expression. This can lead to variability between operators. The accuracy of the AIM assay relies on a well-characterized set of markers, and without understanding the markers thoroughly, it may not identify the complete population of antigen-specific T cells. Overall, the AIM assay is a widely used method for studying antigen-specific T cells, particularly in immunological research.

Applications: The AIM assay is useful in detecting and characterizing rare antigen-specific T cells [42]. Without requiring predefining the specific epitope, it is suitable for novel epitope discovery using proteins or pathogens.

## 4. Emerging Whole-Blood Assays to Measure Beta-Cell Antigen-Specific CD4^+^ T-Cell Responses

**Figure 1:**
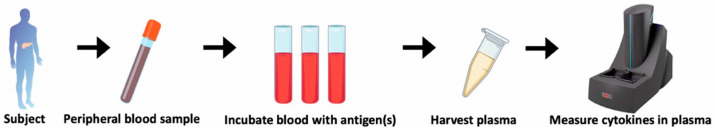
Workflow for a whole-blood assay to measure cytokine production in response to antigen stimulation. Peripheral blood samples are collected from the subject and incubated with specific antigens. After incubation, plasma is harvested, and cytokine levels in the plasma are measured using a highly sensitive platform, such as the V-plex system from Mesoscale Discovery (MSD), which can measure sub-pg/mL concentrations of IL-2.

A new class of T-cell assay, which uses whole blood, has recently been reported [55]. These assays use heparinized whole blood and measure antigen-specific T-cell responses by the cytokines secreted into the plasma. Recently, we have developed a whole-blood assay that measures C-peptide-specific CD4^+^ T-cell responses. We call this the Beta-cell Antigen-Specific T-cell Assay, or BASTA. BASTA measures beta-cell antigen-specific CD4^+^ T-cell responses by the release of IL-2 in response to ex vivo stimulation with relevant peptide autoantigens (Figure 1) [56]. Heparinized whole blood is cultured with the relevant peptide antigens for 24 h. Then, the plasma is collected for analysis. At this point, the plasma can be stored frozen or assayed immediately (Figure 1). After testing a panel of preproinsulin peptides, we found that full-length C-peptide (PI_33–63_) [57] stimulated the most T1D-specific T-cell responses. Using C-peptide, BASTA gave an AUROC of 0.86 when responses between HLA and age-matched individuals with and without T1D were compared [56]. Whole-blood assays for autoimmune T-cell responses, like BASTA, are facilitated by the detection of the very low concentrations of IL-2 secreted by the scarce beta-cell autoantigen-specific CD4^+^ T cells through Mesoscale Discovery (MSD), as previously described in a related assay for T-cell responses associated with celiac disease [55,58].

Advantages: The advantage of whole-blood assays, including BASTA, is their simplicity [56]. Since the assay uses whole blood, no cell isolation or complex processing steps are required. The assay is also logistically simple. The plasma can be stored, and shipped if necessary to a central laboratory, before analysis. Finally, BASTA uses a very small volume of blood; we use 1.0 mL of whole blood per antigen. For each assay, we run a positive and a negative control, which means that the minimum assay only requires 3.0 mL of blood. Additional antigens or antigen mixes can be added or omitted as required. These features make BASTA uniquely well suited to the analysis of CD4^+^ T-cell responses in clinical studies and clinical trials.

Disadvantages: BASTA detects IL-2 secretion in response to an antigen, but it does not provide detailed information about the function of the responding cells [56]. The assay relies on a high-sensitivity electrochemiluminescence IL-2 detection method, which is currently expensive. The assay cannot be used on frozen/thawed PBMCs, which means that it cannot be used on PBMC samples that have been stored from clinical trials or other clinical studies. Consequently, it is not able to be used retrospectively to analyze samples collected from earlier research projects.

## 5. Conclusions and Future Directions

Due to the immunobiology of T1D, measuring antigen-specific T-cell responses continues to be a technical challenge. We now have several assays, as reviewed above, that can detect autoantigen-specific T-cell responses. Recent advances, specifically BASTA, open the possibility of simple and sensitive assays for measuring beta-cell antigen-specific T-cell function. The next challenge is to define the optimal set of peptide antigens that elicit T1D-specific T-cell responses. Despite our expanding knowledge of beta-cell-derived epitopes, many stimulate T-cell responses equally in individuals with and without T1D, which raises questions about the relevance of T-cell responses to these epitopes in the immune pathogenesis of T1D. Determining if an assay can distinguish between samples from people with and without T1D is a simple first step, but there are many other clinical parameters, such as age of onset, islet autoantibodies and insulin requirement, which, together with T-cell assays, will give a more complete picture of the individual’s disease. The assays currently available measure antigen-specific T-cell function. A simple assay that could measure the full breadth of the antigen-specific T cells, i.e., CD4^+^ Teff, CD8^+^ Teff and regulatory T cells, would give a comprehensive view of the autoimmune T-cell responses driving beta-cell destruction, to which we remain largely blind.

## Figures and Tables

**Table 1:** Summary of the main T-cell assays used in T1D.

Assay	Advantages	Disadvantages	Applications
CFSE-based Proliferation Assay [37]	Easy to perform and cost-effective. Quantifies antigen-specific cell proliferation. High sensitivity. Can be modified to include activation markers and cytokine secretion.	Time-consuming to analyze each sample. Only data on proliferation history. Many proliferating cells may be bystanders.	Investigating antigen-specific T-cell activation and proliferation. Isolating viable antigen-specific T cells.
IFNγ ELISpot Assay [40]	Highly sensitive for detecting low-frequency T cells. Quantitative data on cytokine production.	Requires large blood volumes. Labor-intensive and requires optimization. Only gives data on cytokine production.	Identify rare antigen-specific T cells. Measure cytokine production.
pHLA-Tetramer-Based Assay [41]	Highly specific and quantitative. Recoverable for future analysis as long as the cells are alive.	Requires prior knowledge of HLA type and epitopes. Labor-intensive and technically demanding. Custom reagents required.	Precise detection and characterization of antigen-specific T cells with high specificity. Analysis of antigen-specific TCR repertoire.
AIM Assay [42]	Sensitive for rare antigen-specific T cells. Detect responses to a wide array of antigenic targets.	Potential variability due to background responses. Variable data interpretation. Relies on well-characterized markers for accuracy.	Detects and characterizes rare antigen-specific T cells. Suitable for novel epitope discovery.

## Abbreviations

The following abbreviations are used in this manuscript:

HLA	Human Leukocyte Antigen
T1D	Type 1 diabetes
TCR	T-cell receptor
CFSE	5,6-carboxyfluorescein diacetate succinimidyl ester
ELISpot	Enzyme-linked Immuno spot
AIM	Activation-induced marker
